# Hepatic Failure following Metronidazole in Children with Cockayne Syndrome

**DOI:** 10.1155/2020/9634196

**Published:** 2020-01-29

**Authors:** Pedram Ataee, Avat karimi, Kambiz Eftekhari

**Affiliations:** ^1^Liver and Digestive Research Center, Research Institute for Health Development, Department of Pediatric, Kurdistan University of Medical Sciences, Sanandaj, Iran; ^2^Department of Pediatric, Kurdistan University of Medical Sciences, Sanandaj, Iran; ^3^Pediatric Gastroenterology and Hepatology Research Center, Tehran University of Medical Sciences, Department of Pediatric, Bahrami Children's Hospital, Tehran, Iran

## Abstract

Cockayne syndrome is an uncommon autosomal recessive disease characterized by microcephaly, abnormal growth, and pathologic premature aging. The purpose of this report is to evaluate liver failure in children with Cockayne syndrome following metronidazole administration. The first case was a 2-year-old boy with Cockayne syndrome. He had been treated with metronidazole for gastroenteritis. 48 hours after treatment initiation, he was hospitalized due to jaundice, intractable vomiting, and agitation. Unfortunately, he died of acute liver failure. The second case was a 5-year-old boy with Cockayne syndrome as well, who had been treated with amoxicillin and metronidazole for a dental infection. He developed jaundice, drowsiness, lethargy, and anorexia after treatment. At hospital, the child received supportive treatment, and his general condition gradually improved. The liver enzyme levels decreased. He was finally discharged in good general condition. The mortality after metronidazole consumption in patients with Cockayne syndrome due to liver failure is very high. The awareness of the dangers of using metronidazole in these patients is valuable.

## 1. Introduction

Cockayne syndrome is a rare congenital disease inherited as an autosomal recessive pattern. This syndrome was first described in 1936 by EdwardCockayne [1]. Clinical manifestations are short stature, failure to thrive (FTT) in infancy, microcephaly, premature aging (progeria), severe photosensitivity, hearing loss, tooth decay, vision problems, bone malformations, neurodevelopmental disorder (NDD), and moderate to severe delay in learning. These children are highly photosensivite to sunlight, and in some cases, even a small amount of sunlight can cause sunburn or blistering on the skin [2, 3]. Photosensivity is often considered a major diagnostic feature. The syndrome can be classified into three types according to the severity of the disease and the onset of symptoms [4]:

1. Type 1 (or type A) is a classic and moderate form of the disease in early childhood (late first decade) characterized by facial manifestations and physical characteristics [5, 6]. It is also called cerebro-oculo-facio-skeletal (COFS) syndrome or “Pena-Shokeir syndrome type II.”
2. Type 2 (or type B) is a severe or early onset of the disease at birth characterized by facial features and developmental disorders [5, 6].
3. Type 3 (or type C) is the mildest form of the disorder.

Children with type 1 usually have progressive neurological degeneration that can lead to death in the second or third decade of life, while patients with type 2 usually die at the age of 6–7. One of the prominent features of Cockayne syndrome is impaired DNA repair. In this syndrome, the ratio of male to female is equal. Type 1 develops in childhood, whereas type 2 occurs at birth or in infancy and has a worse prognosis. It is estimated that the prevalence of the syndrome in the United States and Europe will affect 2 to 3 per million newborns. These children are usually normal at birth. The average age at death is 8.4 years and rarely survives until the age of 30 [7]. Sun protection by clothing and sunscreen are important medical care for these children. The patients also have a serious reaction to metronidazole so that the use of this drug can lead to liver toxicity and life-threatening liver failure. Its clinical manifestations can be similar to paracetamol poisoning, with significantly increased liver transaminases and important coagulation disorders [6]. Therefore, metronidazole is contraindicated in these patients, and intravenous administration may be fatal [6, 7]. This complication occurs in all patients with Cockayne syndrome following the administration of metronidazole [7]. This drug is extremely safe, however [7]; a few cases of metronidazole-induced liver toxicity have been reported, usually associated with ethanol consumption [8]. In some patients, hepatotoxicity is also reported in the absence of ethanol, which may occur by alternative mechanisms [8], so that metronidazole should be used with extreme caution and careful monitoring of liver function and coagulation state. This control should be continued 2 to 4 weeks after the last dose [7]. We report two cases of Cockayne syndrome that presented with acute liver failure after metronidazole administration, one of them died despite supportive management but the other was discharged in a good general condition. Our aim is to emphasize the dangers of using metronidazole in children with Cockayne syndrome.

## 2. Case Presentation

### 2.1. Case 1

A 2-year-old boy was the known case of type B Cockayne syndrome (based on genetic testing). The patient had been treated with ondansetron and metronidazole for gastroenteritis one week before admission. Forty-eight hours after starting the medication, jaundice, intractable vomiting, and agitation appeared. At the initial examination, he was extremely restless and irritable. The patient was admitted at the pediatric intensive care unit (PICU). Gradually, within a few hours he developed drowsiness and decreased consciousness and responded only to painful provocations. He had no previous history of liver disease. The ultrasound showed increased liver echogenicity, normal intra- and extrahepatic bile ducts, and little free fluid in the pelvis and around the liver and spleen. The patient was treated with cefotaxime and vancomycin, vitamin K, and pantoprazole. Transfusion of fresh frozen plasma (FFP) and packed cells (PC) was performed. The brain CT scan was normal. Viral markers such as HAV, HBV, HCV, CMV, and EBV were negative. The results of paraclinical tests are summarized in Table 1. Unfortunately, the patient died after 48 hours despite conservative treatment.

### 2.2. Case 2

A 5-year-old boy was the known case of type B Cockayne syndrome (based on genetic testing). He had been treated with amoxicillin and metronidazole for a dental infection one week before admission. Three days after the onset of medication, jaundice, drowsiness, lethargy, and anorexia occurred. At the time of admission, he was alert and presented with jaundice, mild hepatomegaly (border of liver was palpated 3 cm below the rib cage), and pitting edema of the lower limbs. The child had a history of elevated liver enzymes at the age of 2 years. At that time, initial evaluation for HBV, HCV, autoimmune hepatitis, and hypothyroid were negative, and muscle enzymes including CPK were normal; therefore, no definitive diagnosis was made. On the other hand, as Cockayne syndrome can cause hepatic involvement, and since no other secondary cause was found for hepatic involvement, this disorder was attributed to Cockayne syndrome. The liver enzymes had reached normal levels with ursodeoxycholic acid (UDCA), and no liver biopsy was performed. The patient was hospitalized and treated with cefotaxime, vitamin K, pantoprazole, PC, FFP, and albumin transfusion. Gradually, his general condition improved, and the INR and liver enzymes decreased. Finally he was discharged after 8 days with UDCA prescription. Assessments for infections including HAV, HBV, HCV, CMV, and EBV were negative. The results of paraclinical tests are summarized in Table 1. The only pathologic finding in the ultrasound was an increase in liver echogenicity. Further evaluations performed one month after discharge depicted liver enzymes 4–5 fold higher than upper limit normal, total bilirubin 15 mg/dl and direct bilirubin 10 mg/dl, and INR 1.2. The treatment with UDCA was continued, and follow-up was continued.

## 3. Discussion

Two cases of liver failure secondary to metronidazole in patients with Cockayne syndrome referred to Besat Hospital in Sanandaj are presented. These cases are reported for the first time in Iran. The first case developed acute liver failure within 48 hours after the onset of metronidazole and unfortunately died. He was not a candidate for liver transplant because of severe mental retardation (MR). The second patient, unlike the first patient, had a previous history of liver disease. He had recently presented with symptoms of acute liver failure. The patient also had severe mental retardation (MR). Fortunately, he improved with supportive management and was discharged in good general condition. He also was able to cope well with acute hepatic failure due to the partial adaptation of the liver secondary to the previous hepatic involvement. Although both patients had acute liver failure at admission (liver enzymes >50 times the normal limit, INR >4), they had different stages of encephalopathy.

Wilson et al. reported 8 cases of acute liver failure following metronidazole in patients with Cockayne syndrome, and three of them died. The interval between admission and death varied between 6 and 11 days [6]. The reported cases of acute liver failure following metronidazole in patients with Cockayne syndrome are very limited worldwide. However, given the high importance and mortality of this drug in patients with this syndrome, it is necessary to inform all physicians, dentists, pharmacists, and healthcare professionals about the dangers of using metronidazole in Cockayne syndrome patients, to prevent similar occurrences.

## Figures and Tables

**Table 1:** The results of initial paraclinical tests at admission to the hospital.

Case	CBC			AST	ALT	ALKP	Bil		Alb	INR	PTT
	WBC	RBC	PLT				T	D			
1	20900	12.2	277000	2535	2815	1265	13.7	9.6	3.3	5.1	45
2	8800	12.6	151000	2598	2248	1654	16.0	11.8	3.0	4.6	42
